# How advances in machine learning drive early detection and risk prediction of early-onset colorectal cancer

**DOI:** 10.3389/fonc.2026.1916796

**Published:** 2026-08-26

**Authors:** Sudha S. Murthy, Anna Schuh

**Affiliations:** 1 Department of Histopathology and Molecular Oncology, Krishna Institute of Medical Sciences (KIMS), Hyderabad, India; 2 Department of Oncology, University of Oxford, Oxford, United Kingdom

**Keywords:** cfDNA fragmentomics, circulating tumour DNA, early cancer detection, early-onset colorectal cancer, liquid biopsy, long-read sequencing, machine learning

## Abstract

Early-onset colorectal cancer (EOCRC), defined as colorectal cancer diagnosed before age 50, is rising across high- and middle-income settings whilst organised screening stays anchored to older age thresholds. Blood-based liquid biopsy, combined with machine learning, is the most plausible route to early detection in this group because it does not depend on bowel preparation, endoscopy capacity, or adherence to stool-based testing. The gap is structural: incidence climbs fastest in the population below the age at which any guideline-endorsed modality is offered. The analytical challenge is that early-stage tumour-derived signals in plasma are low in abundance and distributed across heterogeneous molecular layers: circulating tumour DNA mutations, aberrant methylation, cfDNA fragmentomics, and small non-coding RNA. Machine learning converts these into a single calibrated probability. This review examines where artificial intelligence (AI)-driven liquid biopsy genuinely adds diagnostic value in EOCRC, distinguishes components in which learned models are decorative from those in which they are mechanistically necessary, and identifies the validation deficit separating research cohorts from deployable clinical tools. It summarises the first-generation tools used clinically for early detection and post-treatment monitoring, then considers analytes from exosome-bound microRNAs to long-read whole-genome sequencing of circulating plasma DNA, which reads cytosine modification natively, resolves methylation and fragmentation on single molecules, and characterises structural events short reads cannot anchor. Any analyte can feed a learned model, but more diverse input yields better discrimination. The central argument is that approved, guideline-included blood tests were validated in populations aged 45 and above, and their performance in younger patients cannot be assumed.

## Graphical Abstract

**Figure:**
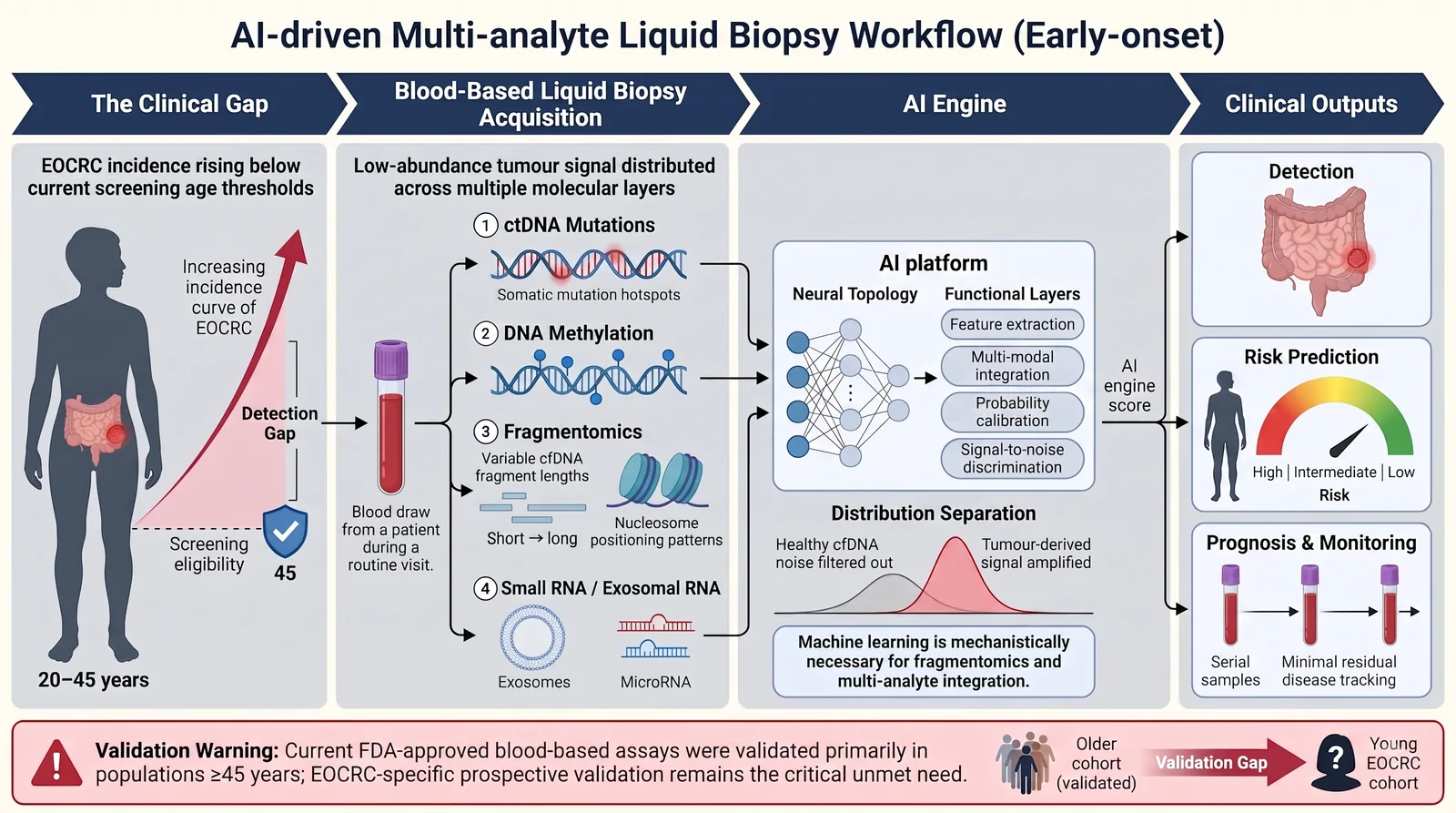
Workflow for AI-driven liquid biopsy in early-onset colorectal cancer (EOCRC). A single plasma draw yields tumour-derived material across four molecular layers, each present at low abundance in early disease: somatic ctDNA mutations, aberrant methylation, cfDNA fragmentomics, and circulating or exosomal RNA. A machine-learning model integrates these layers, suppresses healthy-cfDNA background, and returns a calibrated output for detection, risk stratification, and longitudinal monitoring. The banner records the field’s binding constraint: approved blood-based assays were validated chiefly in cohorts aged 45 and above, and prospective validation in younger EOCRC-specific populations is the outstanding requirement.

## 1 Introduction

Stage at diagnosis decides almost everything in colorectal cancer. Localised disease carries a 5-year survival approaching 90%; once metastatic, that figure falls to roughly 15% (1). For most of the history of CRC screening, the population of concern was older, and the programmes were built accordingly. Early-onset colorectal cancer (EOCRC) has broken that assumption. Incidence in adults under 50 years has climbed for decades, and the disease that presents is not simply late-onset cancer in a younger body: it skews left-sided and rectal, with signet-ring and mucinous histology more common than in older patients. Younger patients also tend to present later; their symptoms are attributed to haemorrhoids or irritable bowel, and nobody is screening them.

The result is a gap that no current programme fills. Colonoscopy defines the screening pathway, but it is expensive and capacity-limited, and the stool-based alternatives lose much of their value to poor adherence. Average-risk reimbursed screening, by faecal immunochemical test (FIT) and/or colonoscopy, begins at 45 or 50 in most high-income healthcare systems. The under-45 group, where the relative rise in EOCRC is steepest, has no organised screening at all (15). Expanding access in this population may need a more convenient approach, a simple blood-based test that can be offered opportunistically during routine healthcare encounters, even when the visit is unrelated to colorectal cancer. Liquid biopsy is the obvious candidate. Whether its signal is usable at the low tumour fractions of early, asymptomatic disease is a question that turns on the analytics, which is where machine learning (ML) enters. Throughout, this review holds a deliberate line between what is clinically validated, what is emerging from external validation, and what remains proof of concept; the distinction is tracked explicitly in the layer-by-layer analysis and in **Table 1**, because in EOCRC, the gap between a promising model and a deployable one is the substance of the problem, not a caveat to it.

**Table 1:** Molecular layers in AI-driven liquid biopsy for EOCRC, ranked by dependence on learned models and by the maturity of EOCRC-specific evidence.

Layer	ML dependence	EOCRC-specific evidence	Status
Methylation	Moderate. Signal dense; classical classifiers competitive	Indirect. Approved tools validated in cohorts aged 45 and above	Deployed (Shield, age 45+) (2–4)
Fragmentomics	High. The signal is only extractable by learned models	Emerging. Multicentre CRC cohorts, not EOCRC-specific	Research and external validation begun (7)
Small/circulating RNA	High. Multi-marker integration via boosted trees	Strong. ENCODER built and tested in EOCRC	Research, prospective trials recruiting (8, 9)
Long-read WGS (native methylation)	High. Single-molecule classifier across genome-wide signal	None EOCRC-specific. Shallow WGS (0.2–1×) for detection; deep for characterisation	Research only, monitoring at research stage; not validated for screening (12, 14).
Clinical/EHR features	Moderate. Tabular ML on real-world data	Direct but retrospective. Under-45 prediction	Research, calibration unestablished (10)

## 2 Why early-stage detection is an analytical problem, not only an assay problem

Tumour fraction governs early detection from blood. In advanced disease, tumour-derived cfDNA makes up enough of the plasma pool that a single marker can find it; in asymptomatic or pre-invasive disease, it does not, and the fraction often sits below the point where any one biomarker class separates cases from controls at both high sensitivity and acceptable specificity (2). The multi-modal design of contemporary screening assays is forced by that biology, not chosen for elegance.

Guardant Shield shows one workable architecture. It is a targeted, methylation-partitioning assay read at high depth (its mechanism is detailed in Section 3.1), reading three orthogonal feature classes across roughly 2,000 cancer-associated regions, somatic mutations, aberrant methylation, and fragmentation, and collapsing them into one binary call (2). In ECLIPSE, a study of average-risk individuals, it reported a CRC sensitivity of approximately 83% at a specificity near 90%, which carried it to Food and Drug Administration (FDA) approval and then into National Comprehensive Cancer Network (NCCN) guidance as a 3-yearly option (3, 4). That number belongs to the integration. No single feature class reaches the same operating point on its own at screening-relevant tumour fractions, which is the point of combining them. The same study also marks where the technology sits today: sensitivity for advanced precancerous lesions was only 13%, and sensitivity for stage I CRC was 65%, against a false-positive rate near 10% (18). The integration recovers a usable cancer signal; it does not yet recover the early and pre-malignant signal that screening most needs. The data volume per patient from a deep targeted panel of this size is modest by genomic standards, and the integration it requires is real but bounded; this is not the regime where ML is mechanistically indispensable so much as useful.

The regime where ML becomes indispensable is the genome-wide one. When the discriminating signal is spread across thousands of fragmentomic features and a scatter of sparse, low-frequency mutations, with no single locus carrying it, no hand-written decision rule integrates it well. A trained model that weights and calibrates those features is what produces a usable probability. The distinction runs through the rest of this review: in some applications, ML sharpens a result simpler methods could approximate; in others, the signal exists only because a model can see it. A targeted panel is the first kind and genome-wide fragmentomics is the second.

## 3 Early detection calls for analysis of multiple molecular layers in the liquid biopsy signal revealed by advanced machine learning

### 3.1 Methylation

Aberrant methylation is the most mature epigenomic signal in CRC. The marks are abundant, stable in plasma, and laid down early in colorectal carcinogenesis. The approved Shield assay is built on these marks. It is a methylation-partitioning cell-free DNA (mp-cfDNA) test in which methyl-binding-domain beads separate methylated from unmethylated fragments by affinity. The two partitions are prepared as separate libraries, and a targeted epigenomic and somatic panel then enriches informative regions for deep NGS (5). The partitioning is physical, not chemical, which sidesteps the bisulfite conversion that fragments and biases cfDNA, but it remains a targeted panel rather than a genome-wide readout. ML matters here, but its role is bounded: models classify the profiles and set the thresholds, and because the signal is dense and the panel targeted, conventional classifiers with careful feature engineering stay competitive. The single-marker era is a caution here: circulating methylated SEPT9, the first CRC methylation marker in wide use, managed sensitivity below 50% in a large prospective cohort (6). It was absorbed rather than replaced, with later assays retaining the principle and adding methylation sites, moving from a single marker to multi-region panels with learned classifications.

### 3.2 cfDNA fragmentomics

Fragmentomics is the layer where ML is not optional. Tumour-derived cfDNA fragments carry a different signature from those shed by healthy tissue in length, in end motif, and in nucleosome positioning, and none of it touches the underlying sequence (2). The signal is diffuse and genuinely genome-wide, weak at any single locus, which puts it out of reach of fixed rules and within reach of a model trained on the aggregate pattern. The field was established by DELFI, which fed the ratio of short to long fragments in megabase windows across the whole genome, from shallow whole-genome sequencing (WGS), to a gradient-boosted classifier for cancer detection and localisation (16)^^1^^. The discrimination there comes from the genome-scale pattern, not from a predefined set of regions, which is what separates it from a targeted panel. A 2025 multicentre study extended the approach with features including the differential representation of Alu elements and long terminal repeats across training, internal, and external validation cohorts (7), and the same signal tracks tumour burden over treatment: DELFI-TF estimates tumour fraction from genome-wide fragmentation without prior knowledge of tumour mutations, validated in colorectal and lung cohorts (17). The deeper point is that fragment length and geometry carry diagnostic information independent of any variant call. That independence becomes decisive when the event you are hunting is a structural rearrangement, the kind of thing short fragments represent poorly and read length captures directly.

### 3.3 Small non-coding and circulating RNA

Circulating microRNA and other small RNA species carry the strongest EOCRC-specific evidence, from the one programme built for this population rather than borrowed from an older cohort. ENCODER, a multicentre study across four countries, identified six biomarkers by small RNA sequencing, two cell-free and four exosome-based miRNAs; trained an extreme gradient boosting model on RT-qPCR readouts; and tested it in an independent external case–control cohort (8). The discrimination was high, with an area under the receiver operating characteristic (ROC) curve of 97.5% in training and 95.6% in testing, holding at 98.5% in those aged 20 to 35 (8). The figures that matter for screening are the stage-specific ones: sensitivity for stages I to III was 97.3%, and sensitivity for high-grade dysplasia was 61.5%, at 87.5% specificity (8). That precancer sensitivity is high for a blood-based assay and above the single-digit figures reported for approved methylation-based tests, though the comparison is indirect: ENCODER is case–control, which tends to overstate sensitivity relative to the consecutive screening cohorts in which those tests were evaluated. The authors attribute part of the assay’s stability across ages to methylation signal being more age-dependent. What counts is the provenance: the model was developed in an EOCRC cohort, not fine-tuned from an average-risk dataset, which speaks to the validity problem this review keeps returning to. Circular RNA and exosome approaches built for EOCRC are now recruiting prospectively (9).

### 3.4 Long-read single-molecule whole-genome sequencing

Long-read whole-genome sequencing (LRS), on Oxford Nanopore and PacBio, sits apart from the other layers because it changes what can be measured, not just how the measurement is modelled. Three properties bear on EOCRC. It reads cytosine modification natively, in the same pass as the base, with no bisulfite or enzymatic conversion; conversion pays in GC skew, amplification bias, and alignment artefacts, and bisulfite collapses 5mC and 5hmC into one signal (13, 14). Pulling 5hmC apart matters most at the early stage, where the shifts are slight and the combined signal hides which modification is moving (13). Read length then captures the full cfDNA fragment-length distribution, including the longer fragments short-read protocols clip, so fragmentomic and nucleosome features are read off the molecule rather than reconstructed from its pieces (14), and a single read spans repetitive or structurally complex loci that short reads cannot anchor, which is exactly where the discriminating event is a structural variant or buried in low-mappability sequence.

The sharpest demonstration of what this buys in detection is single-molecule methylation classification. A nanopore approach built cfDNA methylomes and trained a per-read classifier to call each molecule as tumour or immune-in-origin, carried through to longitudinal CRC monitoring in a research setting (12). The clinical caveat matters: long-read sequencing is not used for CRC monitoring in practice today. Deployed minimal-residual-disease assays are tumour-informed targeted panels, typically UMI-duplex chemistry, that track mutations identified at diagnosis, and they remain under evaluation in CRC trials. That tumour-informed design achieves a sensitivity far above what any tumour-agnostic early-detection assay can reach, because it knows what to look for. A separate nanopore study found cancer-derived DNA detectable at shallow coverage as low as 0.2×, with methylation, fragmentation, and copy-number features broadly concordant with short-read WGS and bisulfite data (14). That concordance is the reassuring finding: long reads are not measuring something different; they are measuring the same thing on intact single molecules. Thus, the honest scope of LRS in EOCRC is signal resolution, single-molecule classification, and research-stage monitoring. No one has validated LRS-based plasma screening in an EOCRC population, and no approved EOCRC screening assay runs on long reads today. The case for LRS is mechanistic and forward-looking. It is not a claim that long reads screen better now.

Depth is the variable that decides which of these capabilities is on the table, and the two do not coexist in one cheap assay. Shallow WGS, in the 0.2× to 1× range, is what makes genome-wide long-read signal economically conceivable at screening scale: it delivers the aggregate readouts a classifier needs, copy-number profile, fragmentomics, and methylation density at a per-sample cost that 30× sequencing of asymptomatic young adults could never justify (14). What it gives up is depth-dependent: reliable per-base variant calling, structural-variant breakpoint resolution, and paralog disambiguation. The coherent role for long reads in EOCRC is therefore split by depth: shallow WGS for detection, feeding methylation and fragmentomic features into the model, and deep sequencing reserved for characterisation of the positives. For that characterisation step, the diagnostic tissue is usually available, and conventional genotyping of the biopsy resolves breakpoints and structural detail directly; deep plasma WGS becomes the relevant route mainly when tissue is insufficient or when the structural question is one tissue genotyping does not answer.

## 4 From detection to risk prediction and prognosis

Risk prediction in EOCRC is really two questions, and they should not be run together. The first is who, amongst young people, warrants earlier or more intensive evaluation before any cancer is suspected. The useful data here are clinical rather than molecular, and ML models trained on electronic health record features have predicted EOCRC below screening age, surfacing risk factors and reaching discrimination good enough to inform earlier workup, though not yet to direct it (10). These features sit alongside the established axes of stratification: polygenic risk scores, family history, lifestyle exposures, and comorbidities such as inflammatory bowel disease and other autoimmune conditions, any of which could enrich a young population to the prevalence at which blood-based screening becomes cost-effective. The structural catch is that these models learn from retrospective real-world data in which EOCRC is rare, and how they calibrate in a live screening population is unknown.

The second question comes after diagnosis: who recurs, and who survives? This is a different problem from screening, and an easier one for liquid biopsy, because post-diagnosis surveillance can be tumour-informed: the recurrence assay looks in follow-up samples for the specific alterations catalogued at diagnosis, which yields a sensitivity far above what tumour-agnostic early detection can reach. The ENCORE multicentre study pushed the circulating-biomarker approach into prediction of long-term recurrence-free and overall survival in EOCRC specifically, showing that the same class of liquid biopsy signal carries prognostic weight in this population, not only diagnostic weight (11). That reframes liquid biopsy as something used across the whole disease course rather than at a single screening moment, and the framing will most likely justify the cost of deployment in a younger cohort, where survivorship is longer and the surveillance window stretches over decades.

## 5 The validation gap

Three questions sit underneath any serious attempt to deploy these assays in a young population, and the barriers section returns to each: whether 2 mL of plasma holds enough tumour-derived molecules at early-stage fractions, whether screening can be cost-effective at low prevalence, and whether the efficient route is a single-cancer or multi-cancer assay. The prior question, and the one this section is about, is narrower and more often skipped: Whom were these models trained on?

The most important limitation in this field is also the one least likely to come up at the point of care. Every blood-based screening assay with regulatory approval and guideline inclusion was developed and validated in average-risk populations aged 45 and older (2–4). ECLIPSE, the cohort that set Shield’s numbers, starts at 45, and so does the indication. EOCRC is not a late-onset disease moved younger: it differs in anatomical distribution, in histological subtype, in methylation landscape, and in microbial associations. A classifier tuned to separate tumour signal from background in a 60-year-old need not behave the same way in a 35-year-old, where tumour fraction, fragmentation background, and the field of competing benign signals may all be different. The assumption that performance transports is rarely stated out loud and almost never tested where it would actually be applied.

Confirmation bias does its work quietly here. The headline figures from the approved assays get read as proof that blood-based detection works, and the conclusion is then extended to EOCRC by implication, without anyone quite deciding to do so. The EOCRC-specific evidence that exists (ENCODER and ENCORE) runs on different analytes, sits in research cohorts, and has no regulatory standing or prospective screening validation (8, 11). Put plainly, the tools that are deployed were not validated for the population this review is about, and the tools built for that population are not deployed. Treating the two as one body of evidence makes the problem look closer to solved than it is.

## 6 Barriers to clinical translation and outlook

The barriers between current performance and clinical utility in EOCRC are concrete. Advanced adenoma is the first problem: the lesion whose removal actually prevents cancer is detected weakly by blood-based assays relative to their cancer sensitivity, which blunts the preventive case for screening as opposed to the diagnostic one. Shield’s 13% APL sensitivity in ECLIPSE sits against roughly 43% for multi-target stool DNA, and on cost-effectiveness analysis, stool testing and colonoscopy are both more effective and less costly than the blood test, which is cost-effective only relative to no screening at all (18). Its advantage is adherence, not accuracy: offering a blood draw raised screening uptake from 13% to 31% in one trial (18), which is the case for it in a population that will not otherwise be screened. Generalisability compounds both. Across ancestry, geography, and assay platform, most EOCRC-specific classifiers are unproven, and the small RNA and fragmentomic signals are fussy about pre-analytical handling in ways that make multi-site deployment harder than it looks on a single-centre ROC curve. The failure mode is specific: models tuned on a development cohort routinely lose calibration when moved across ancestry, assay platform, and site, and the drop is largest for the diffuse fragmentomic and small-RNA signals whose feature distributions shift with pre-analytical handling. Reported single-centre performance is therefore an upper bound, not an estimate of what a multi-site screening programme would deliver.

Specificity at population scale sits underneath all of these. In a young, low-prevalence population, a specificity near 90% still throws off enough false positives to drive a wave of downstream colonoscopies and a good deal of anxiety, and positive predictive value sinks as prevalence falls. Two further constraints deserve to be stated directly. The first is physical: Shield runs on roughly 2 mL of plasma, and at the tumour fractions of early, asymptomatic disease, the absolute number of tumour-derived molecules carrying any given CRC-specific signal in that volume is small. There is a floor set by sampling statistics that no classifier can argue past. The second is strategic: whether the efficient path is a single-cancer EOCRC test or a multi-cancer early-detection assay that amortises the same blood draw across several tumours is unresolved, and enrichment by risk stratification may be a precondition for cost-effectiveness rather than a refinement of it.

Interpretability binds harder in screening than in most clinical ML. The most sensitive multi-omic detectors are gradient-boosted ensembles or deep neural networks evaluating thousands of genome-wide parameters, and they return a single opaque probability with no locus a clinician can point to. That is tolerable for a validated deployed assay but not for a model still seeking it, where without feature attribution a clinician cannot tell a genuine early-stage signal from a benign confounder such as clonal haematopoiesis of indeterminate potential (CHIP) or localised inflammation, and regulators cannot audit the call. Per-read classifiers that expose which molecules drive a positive are the practical route to that transparency.

The way forward is clear: prospective validation of EOCRC-specific classifiers in age-appropriate cohorts is the rate-limiting step, and the recruiting exosome and circular RNA trials may be the right vehicles for it (9). The architecture most likely to reach the specificity that low prevalence demands is multi-analyte integration, methylation, fragmentomics, and circulating RNA inside one learned model, because errors that are uncorrelated across layers are what drive the false-positive rate down. Long-read WGS is a natural substrate for that integration, since methylation, fragment length, and structural signal come off the same molecules in one assay instead of being stitched together from separate conversion-dependent workflows. Run shallow, at the depth where genome-wide methylation and fragmentomic signal survive; it is also the version of long-read sequencing cheap enough to contemplate at population scale, with deep sequencing held in reserve for the positives. Whether that helps EOCRC discrimination in particular is still an open question; the recruiting cohorts were not designed to answer it. The principle worth holding onto is that a blood test for a 35-year-old has to clear a higher specificity bar than the same test for a 60-year-old, simply because it operates against a lower prevalence. Until the classifiers are shown to be calibrated in the population they are meant to serve, artificial intelligence (AI)-driven liquid biopsy in EOCRC is a strong direction, not yet a standard of care.

The deployed pathway matters more than the stand-alone ROC. The realistic entry point is opportunistic case finding, a test offered during an unrelated primary care encounter and tuned for specificity to triage positives to colonoscopy, with a risk-stratified variant that pre-selects a higher-prevalence subgroup as the alternative. Multi-modal AI has followed this opportunistic-screening logic in other diseases at a population scale (19) and is the credible template for EOCRC once age-appropriate validation exists, judged against colonoscopy uptake and interval-cancer rates rather than laboratory sensitivity alone.

## 7 Conclusion

AI-driven liquid biopsy is the most credible route to closing the EOCRC detection gap, and in the layers that carry the most early-stage signal, fragmentomics, and multi-marker RNA integration, ML is doing necessary work rather than ornamental work. The field has produced two real things: an approved, guideline-included blood test and a separate EOCRC-specific research programme with strong reported discrimination. These are read together too often when the approved tools are validated only for ages 45 and up and the EOCRC-specific tools are not yet in clinical use. What closes the gap is prospective validation of age-appropriate, multi-analyte classifiers in the under-50 population, judged against a specificity standard that respects how low the prevalence really is. The technology is plausible. The validation in the population that matters is the term still missing from the equation.

## Footnotes

1 DELFI Diagnostics. Fragmentomics Platform. Available at: https://delfidiagnostics.com/technology/fragmentomics/ (accessed 6 June 2026).
