# Supplementary material for: How advances in machine learning drive early detection and risk prediction of early-onset colorectal cancer
Source: Front Oncol. 2026 Aug 26;16:1916796. doi: 10.3389/fonc.2026.1916796 (PMC13556045; doi:10.3389/fonc.2026.1916796)
Supplement: Supplementary file 1 [file DataSheet1.pdf]

Supplementary Material 1: Full text prompt used to generate the graphical abstract.

Create a high-resolution, publication-quality scientific graphical abstract titled "AI-driven Multi-analyte Liquid Biopsy Workflow (Early-onset)" at 2126 px width, 16:9 landscape aspect ratio. The aesthetic should be clean, sterile, and vector-based with a standardized clinical palette: deep navy headers, soft slate-grey panel backgrounds, teal accents, and crimson red warning highlights against an off-white/sterile background. All typography uses a crisp, uniform sans-serif font (e.g., Helvetica or Inter). British English spelling ("tumour") is maintained throughout.

The graphic is structured into a top process header, four main horizontal workflow panels with subtle vertical separators, and a full-width bottom validation banner.

---

1. TOP PROCESS HEADER
A continuous horizontal header of four interconnected dark navy chevron banners with bold white text:

1. "The Clinical Gap"
2. "Blood-Based Liquid Biopsy Acquisition"
3. "AI Engine"
4. "Clinical Outputs"

---

2. PANEL 1: The Clinical Gap (Left Column)
- Header Text: "EOCRC incidence rising below current screening age thresholds"
- Anatomical Subject: Left-facing solid dark-slate silhouette of a young adult labeled "20–45 years" at the base, displaying an anatomical colon with a highlighted crimson focal tumour.
- Trajectory Curve: A steep, upward-curving crimson exponential vector arrow labeled "Increasing incidence curve of EOCRC".
- Baseline & Gap: A horizontal dashed line labeled "Screening eligibility" terminating at a blue shield icon with a white checkmark labeled "45". A vertical bracket and soft pink shaded area between the curve and the baseline is labeled "Detection Gap".

---

3. PANEL 2: Blood-Based Liquid Biopsy Acquisition
- Header Text: "Low-abundance tumour signal distributed across multiple molecular layers"
- Acquisition: An arrow from Panel 1 points to a medical purple-top blood collection tube labeled "Blood draw from a patient during a routine visit."
- Four Demarcated Molecular Pathways branching neatly from the tube:
  1. "① ctDNA Mutations": A canonical DNA double helix showing distinct red highlighted somatic mutation hotspots.

2. "② DNA Methylation": A DNA double helix decorated with blue circular lollipop methylation tags.
3. "③ Fragmentomics": Labeled with "Variable cfDNA fragment lengths" (short vs long fragments) and "Nucleosome positioning patterns" (histone core octamers wrapped with cfDNA).
4. "④ Small RNA / Exosomal RNA": Bilayered circular vesicles labeled "Exosomes" and short hairpin duplexes labeled "MicroRNA".

---

4. PANEL 3: AI Engine (Centrepiece)
- Convergence: All four analyte streams feed directly into a central rounded card titled "AI platform".
- Neural Topology (Left half of box): Interconnected multilayer artificial neural network diagram with distinct input, hidden, and output nodes in blue and light slate.
- Functional Layers (Right half of box): Four stacked rounded rectangular pill tags reading top-to-bottom:
  - "Feature extraction"
  - "Multi-modal integration"
  - "Probability calibration"
  - "Signal-to-noise discrimination"
- Clean Space: The region directly above the AI platform box is completely empty and uncluttered.
- Distribution Separation (Directly beneath the AI platform box): A dual probability density curve:
  - Left grey distribution tail labeled "Healthy cfDNA noise filtered out"
  - Right elevated crimson peak labeled "Tumour-derived signal amplified"
- Mechanistic Callout Box: A rounded pale-blue card below the curve reading: "Machine learning is mechanistically necessary for fragmentomics and multi-analyte integration."
- Output Vector: A black arrow emerging from the right of the neural platform labeled "AI engine score".

---

5. PANEL 4: Clinical Outputs (Right Column)
The "AI engine score" vector branches into three vertically stacked modular cards:
1. Detection: Labeled "Detection" with a detailed vector illustration of the human colon and rectum showing a localized, highlighted early-stage tumour.
2. Risk Prediction: Labeled "Risk Prediction", displaying a patient silhouette beside a semicircular gauge arc transitioning smoothly from Red (left) to Yellow (middle) to Green (right) with a needle pointing to the intermediate zone. No text on the arc. Directly beneath the arc, three flat horizontal labels in a single row: "High" | "Intermediate" | "Low", with the word "Risk" centered underneath.

3. Prognosis & Monitoring: Labeled "Prognosis & Monitoring", showing a horizontal timeline tracking three serial purple-top blood tubes labeled "Serial samples" and "Minimal residual disease tracking".

---

6. BOTTOM BANNER: Validation Warning
- Layout: A full-width rose/light-crimson banner with a solid dark-crimson border.
- Left Anchor: Red warning triangle icon "⚠️ Validation Warning" followed by the sentence: "Current FDA-approved blood-based assays were validated primarily in populations ≥45 years; EOCRC-specific prospective validation remains the critical unmet need."
- Right Demographic Bridge: Icon group of multiple figures labeled "Older cohort (validated)" → broad crimson gradient arrow labeled "Validation Gap" → single silhouette badge with a white question mark labeled "Young EOCRC cohort".

TECHNICAL & RENDER SPECIFICATIONS:
- Width: 2126 px | Aspect Ratio: 16:9 | High DPI vector clarity.
- Zero orphaned labels, zero garbled text strings, zero curved path distortions.
- Consistent British English ("tumour", "multi-modal").
